# Oral Health Status, Oral Hygiene, and Behavioural Factors Among Disadvantaged Children: A Cross-Sectional Comparison Between the Peruvian Amazon and Valencia, Spain

**DOI:** 10.3390/dj14070459

**Published:** 2026-07-21

**Authors:** Lucía Miralles-Jordá, Julián Espinosa-Giménez, Anna Paradowska-Stolarz, Mónica Fernández-Mafé, María Dolores Gómez-Adrián, María Teresa Murillo-Llorente, Marcelino Pérez-Bermejo, María Ester Legidos-García

**Affiliations:** 1 Oral Surgery Unit, Department of Dentistry, School of Medicine and Health Sciences, Catholic University of Valencia, 46007 Valencia, Spain; lucia.miralles@ucv.es (L.M.-J.); julian.espinosa@ucv.es (J.E.-G.); monica.fernandez@ucv.es (M.F.-M.); mariadolores.gomez@ucv.es (M.D.G.-A.); 2 Division of Dentofacial Anomalies, Department of Orthodontics and Dentofacial Orthopedics, Wroclaw Medical University, Krakowska 26, 50-425 Wroclaw, Poland; anna.paradowska-stolarz@umw.edu.pl; 3 SONEV Research Group, School of Medicine and Health Sciences, Catholic University of Valencia, 46007 Valencia, Spain; mt.murillo@ucv.es (M.T.M.-L.); ester.legidos@ucv.es (M.E.L.-G.)

**Keywords:** dental caries, oral health inequalities, children, socioeconomic factors, oral hygiene, toothbrushing, Amazon, Spain, health systems

## Abstract

**Background/Objectives**: Dental caries remains a major public health problem in childhood and disproportionately affects socioeconomically disadvantaged populations. Although toothbrushing and oral hygiene are commonly promoted as key preventive behaviours, their protective effect may be limited by structural factors such as access to dental care, fluoride exposure, and preventive services. This study aimed to compare dental caries experience, oral hygiene status, toothbrushing frequency, and sugar consumption among disadvantaged children from the Peruvian Amazon and Valencia, Spain, and to explore whether differences in hygiene behaviours were reflected in caries outcomes. **Methods**: An exploratory cross-sectional school-based study was conducted among 291 children aged 5–12 years attending three purposively selected schools serving socioeconomically disadvantaged communities: one rural school in the Peruvian Amazon (*n* = 162) and two urban schools in Valencia, Spain (*n* = 129). Dental caries experience was assessed using a combined global caries score derived from the dmft and DMFT (CAOD/cod) indices according to dentition type, and oral hygiene status was evaluated using the Simplified Oral Hygiene Index (OHI-S). Toothbrushing frequency and sugar consumption were collected through structured questionnaires. Group comparisons, Spearman correlation analyses, and exploratory multiple linear regression models were performed. **Results**: Mean global caries scores did not differ significantly between Peruvian and Spanish children (3.71 ± 2.86 vs. 4.07 ± 3.44; *p* = 0.596). However, severe caries experience (score ≥ 8) was more frequent among Valencian children (21.7% vs. 10.5%; *p* = 0.014). Peruvian children showed significantly better oral hygiene status (OHI-S-derived score: 1.25 ± 0.79 vs. 1.49 ± 0.84; *p* = 0.014) and higher toothbrushing frequency (*p* < 0.001). Frequent sugar consumption was similarly high in both groups. Within each population, poorer oral hygiene and lower toothbrushing frequency were associated with higher caries experience in bivariate analyses, although the exploratory multivariable models showed a more limited pattern of association. **Conclusions**: Among the participating schools, children from the Peruvian Amazon showed better oral hygiene indicators and more frequent toothbrushing than Spanish children, while no statistically significant difference in caries experience was detected. Because structural variables such as fluoride exposure, dental attendance, and access to restorative care were not directly measured, explanations involving healthcare infrastructure should be considered as candidate hypotheses to be tested in future, adequately powered and adjusted studies, rather than as confirmed mechanisms. Findings should not be generalised beyond the participating schools.

## 1. Introduction

Dental caries is a multifactorial, biofilm-mediated disease characterized by the progressive demineralization of dental hard tissues due to acidic by-products generated by bacterial fermentation of dietary carbohydrates [1]. It remains the most prevalent chronic disease in childhood worldwide and represents a significant global public health concern, affecting an estimated 2.3 billion people globally [2]. The disease is particularly burdensome in low- and middle-income countries (LMICs), where limited access to preventive services and restorative care results in high levels of untreated decay, pain, and associated quality-of-life impairment [3].

The etiology of dental caries involves a complex interplay of biological, behavioral, socioeconomic, and environmental factors [4]. According to the classical Keyes model, the simultaneous presence of cariogenic microorganisms, a susceptible tooth surface, and fermentable carbohydrates is necessary for disease development [5]. However, more recent conceptual frameworks have emphasized the critical role of social determinants of health, including socioeconomic status, access to dental care, exposure to fluoride, and broader structural inequalities [6,7]. The bio-psychosocial model proposed by Peres et al. positions dental caries as a disease deeply embedded in social and commercial determinants, challenging purely behavioral explanations of oral health disparities [2].

Children from socioeconomically disadvantaged backgrounds are disproportionately affected by dental caries. Limited access to preventive dental services, reduced parental oral health literacy, and suboptimal dietary patterns collectively contribute to increased caries prevalence in vulnerable populations [8,9]. Multiple systematic reviews and international studies have demonstrated a strong, consistent association between low socioeconomic status and higher rates of dental caries across diverse geographic and cultural contexts [6,10,11]. In Latin America and Caribbean countries, dental caries has been identified as a marker of social disadvantage, with prevalence rates remaining moderate to high despite global declines [12].

However, socioeconomic disadvantage alone does not fully explain variations in oral health outcomes. Environmental and contextual factors (including healthcare system organization, water fluoridation policies, geographic accessibility of dental services, and cultural practices) may substantially influence disease patterns, even among populations with similar economic conditions [2,13]. Children residing in rural or isolated regions, such as the Peruvian Amazon, may experience fundamentally different exposures and protective factors compared to those in urban European settings, despite sharing socioeconomic vulnerability [14]. The Peruvian Amazon has experienced persistent poverty rates affecting up to 36% of the population, with significant implications for health outcomes and healthcare access [15]. In contrast, children attending “singular schools” in Valencia represent a population at risk of social exclusion within a high-income country with a publicly funded healthcare system [16].

The relationship between oral hygiene behaviors and dental caries has been extensively studied, yet important questions remain regarding the relative effectiveness of individual-level behavioral interventions versus population-level structural interventions [17,18]. While toothbrushing with fluoride toothpaste and good oral hygiene practices are well-established protective factors [19], their impact may be modulated by contextual variables including access to professional dental care, exposure to community fluoride programs, and the availability of restorative treatments [20]. Several longitudinal studies have demonstrated that oral health behaviors in childhood are shaped by structural socioeconomic factors including household income, maternal education, and social capital, suggesting that behavior-focused interventions may have limited effectiveness in the absence of supportive structural conditions [21,22].

The present study was designed to examine these complex relationships by comparing caries experience, oral hygiene status, and associated behaviors in two socioeconomically disadvantaged child populations living in markedly different geographic, cultural, and healthcare contexts: the Peruvian Amazon and Valencia, Spain. By examining children attending three schools officially classified as serving socioeconomically disadvantaged communities, but located in markedly different rural/urban and healthcare contexts, this study aims to describe oral health status, hygiene indicators, and behavioural factors within these school settings and to generate hypotheses about contextual influences on childhood oral health.

## 2. Materials and Methods

### 2.1. Study Design

This study was designed as a cross-sectional, observational, and analytical investigation conducted between March 2025 and November 2025 (clinical examinations: April 2025–July 2025 in the Peruvian Amazon and September 2025–October 2025 in Valencia). The study protocol followed the Strengthening the Reporting of Observational Studies in Epidemiology (STROBE) guidelines for cross-sectional research.

### 2.2. Study Population and Setting

A total of 291 children aged 5–12 years were included in the study. Participants were recruited from three educational institutions serving socioeconomically disadvantaged populations across two contrasting geographical settings. The Peruvian Amazon group comprised 162 children from a rural school in the Requena province (Loreto region, Peruvian Amazon), an area characterized by geographic isolation, limited healthcare infrastructure, and high poverty rates. The Valencian group comprised 129 children from two “singular schools” (colegios singulares) located in marginalized urban areas of Valencia, Spain (Colegio Madre Petra (*n* = 64) and Colegio Santiago Apóstol (*n* = 65)), both of which serve populations at high risk of social exclusion within the Spanish public education system.

The participating schools were purposively selected on the basis of previous institutional collaboration with the research team and their official classification as schools serving socioeconomically disadvantaged populations. No sampling frame of all eligible schools in the Peruvian Amazon or Valencia was constructed, and no random selection of schools was performed. No additional eligible schools were formally invited and declined participation. School-level classification by national education authorities was used as a proxy for socioeconomic disadvantage; no individual-level data on household income, parental education, housing conditions, dental insurance, migration status, previous dental attendance, or access to dental care were collected, so direct individual-level socioeconomic comparability between the two populations cannot be established. In addition, the comparison contrasts one rural school in the Peruvian Amazon with two urban schools in Valencia, so country, school, urban/rural setting, and local healthcare context are structurally confounded. Information on school-level oral-health programmes, water source and fluoridation status, recent preventive campaigns, and routine access to dental services was not systematically collected. The two groups should therefore be understood as children attending three disadvantaged school settings within their respective contexts rather than as strictly equivalent or representative Peruvian and Spanish populations.

### 2.3. Inclusion and Exclusion Criteria

The inclusion criteria were as follows: children aged between 5 and 12 years; enrollment in one of the participating schools; signed informed consent provided by parents or legal guardians; and the physical and cognitive ability to cooperate with the oral examination. Children were excluded if they presented systemic conditions affecting dental development, failed to cooperate during the clinical examination, or had incomplete data for the primary study variables. All eligible children enrolled in the participating schools during the data-collection period were invited to participate through informed-consent letters distributed by school staff to parents or legal guardians. In the Peruvian Amazon school, 178 children were enrolled and invited; 12 did not return a signed consent form and 4 were excluded for incomplete clinical or behavioural data, leaving 162 children (participation rate: 91.0%). In the two Valencian schools, 152 children were invited; 18 did not return a signed consent form and 5 were excluded for incomplete data, leaving 129 children (participation rate: 84.9%). No child meeting the inclusion criteria was excluded on the basis of systemic disease in either setting. A formal a priori sample-size calculation was not performed because the study was designed as a school-based comparison of all eligible children in the participating institutions; this is acknowledged as a limitation, and the post hoc power for the between-group comparison of caries scores is reported in the Limitations section. Final analytic numbers by Valencian school were 64 children from Colegio Madre Petra and 65 from Colegio Santiago Apóstol. Refusals and exclusions in Valencia were recorded for the two schools combined rather than separately by school, which limits the ability to compare participation patterns across individual schools.

### 2.4. Variables and Measurement Instruments

The primary outcome variable was caries experience, assessed using a global caries score derived from dmft and DMFT components according to dentition type. DMFT refers to decayed, missing due to caries, and filled permanent teeth, whereas dmft refers to the corresponding index for primary teeth. CAOD/cod are the Spanish-language equivalents used in the original data collection form. Because the sample included primary, mixed, and permanent dentitions, the term “global caries score” is used throughout the manuscript for the combined outcome. Examinations were performed by calibrated dental examiners following standardized WHO criteria under artificial light, with the child seated and using sterile dental mirrors (Brillant, Hager & Werken GmbH & Co. KG, Duisburg, Germany) and WHO periodontal probes (Hu-Friedy Mfg. Co., LLC, Chicago, IL, USA). Teeth were not professionally cleaned or air-dried before examination; visible debris was removed with gauze or cotton rolls when necessary. Non-cavitated white-spot lesions and doubtful lesions were not recorded as decayed. Teeth missing because of normal exfoliation, orthodontic extraction, congenital absence, or reasons other than caries were not scored as missing due to caries. We acknowledge that combining dmft and DMFT into a single composite score is methodologically debatable, because primary and permanent dentitions differ in number of teeth at risk, eruption status, and susceptibility, and a combined score may obscure differences in untreated versus treated disease across age groups. We retained the composite score because tooth-level data allowing for separate reporting of dmft and DMFT by age and dentition type were available only for the Peruvian sample; for the Valencian sample only the overall caries count was recorded. This data asymmetry is acknowledged as an important limitation and is further discussed in the Limitations section.

Two secondary clinical variables were recorded. Oral hygiene status was evaluated using the Simplified Oral Hygiene Index (OHI-S), based on plaque and calculus accumulation on selected index tooth surfaces, with age-appropriate substitutions when index permanent teeth were absent. Following the Greene and Vermillion framework, oral hygiene was assessed on selected index tooth surfaces, with each surface scored on a 0–3 ordinal scale according to debris and calculus accumulation. For the analyses, an OHI-S-derived oral hygiene score ranging from 0 to 3 was used, with lower values indicating better oral hygiene. In primary or mixed dentition, age-appropriate primary teeth (55, 51, 65, 71, 75, and 85) were substituted when corresponding permanent teeth had not yet erupted. The oral hygiene score was categorized as good (0.0–0.6), fair (0.7–1.8), or poor (1.9–3.0) [23]. The type of dentition (primary, mixed, or permanent) was recorded for the Peruvian sample only; for the Valencian sample, only age and total caries count were available at the tooth-level resolution required to assign dentition category, which precluded stratified or dentition-adjusted between-country comparisons.

Behavioral variables were assessed using brief structured questionnaires administered in Spanish on the day of the clinical examination. Toothbrushing frequency was recorded as the number of times the child brushed per day and categorized as never (0), once daily (1), twice daily (2), or three or more times daily (3+). Sugar consumption was assessed through a locally adapted ordinal frequency questionnaire that captured the habitual intake of sugar-containing foods and beverages on a typical day and was categorized as low/moderate intake (≤1/day) or high intake (>1/day). Toothbrushing frequency was captured as a single ordinal variable; data on fluoride toothpaste use, fluoride concentration, parental supervision, brushing duration, brushing technique, timing of brushing, toothbrush replacement, or interdental cleaning were not collected. A single 24 h dietary recall was used as a complementary tool to support classification of sugar exposure, but it was not used to estimate absolute nutrient intake and, on its own, may not adequately reflect habitual free-sugar exposure. Behavioural questions were answered by the child and were corroborated by parents or guardians when available; however, the proportion of child-only versus parent-assisted responses was not systematically recorded. All forms were administered in Spanish, the language used for school communication in the participating centres. The English versions of the questionnaire items and of the ordinal sugar-frequency scoring system are provided as Supplementary Materials (Supplementary File S1).

Finally, the sociodemographic variables comprised age (in years) and sex (1 = male, 2 = female).

### 2.5. Data Collection Procedures

Data were collected using a standardized clinical record form developed for this study. Clinical examinations were performed by two calibrated dental examiners, both qualified dentists: one examiner operated in the Peruvian Amazon site and one in the Valencian sites. Before fieldwork, both examiners underwent joint training and calibration against a senior reference examiner with previous experience in epidemiological oral-health surveys. Calibration included theoretical training, case-based discussion using clinical photographs and study models, and a pilot clinical session with 15 volunteer children outside the study sample. Examiner reliability was assessed using Cohen’s kappa coefficient and was reported separately for intra-examiner and inter-examiner agreement. Intra-examiner reliability, based on repeat examination of approximately 10% of children at the end of each examination session, yielded kappa values of 0.91 for caries detection and 0.88 for OHI-S scoring. Inter-examiner reliability against the reference examiner yielded kappa values of 0.86 for caries detection and 0.82 for OHI-S scoring. Formal blinding of examiners to behavioural questionnaire responses and formal longitudinal drift assessment across the full fieldwork period were not documented; these limitations are acknowledged in Section 4.8.

Clinical examinations were performed in school settings under standardized conditions: children seated in an upright position, under artificial light (LED headlamp), using sterile dental mirrors (No. 5) and WHO periodontal probes. No radiographic examinations were performed.

Behavioral data were collected through structured interviews conducted with children and supplemented by parental questionnaires when available. Dietary information was obtained using a modified 24 h dietary recall adapted for children.

### 2.6. Statistical Analysis

Data were analyzed using SPSS software (version 26.0; IBM Corp., Armonk, NY, USA). The significance level was set at *p* < 0.05, and 95% confidence intervals were reported. Missing data were not imputed. After applying the exclusion criteria described in Section 2.3, one additional missing value remained for sugar consumption in the Valencian group; therefore, analyses involving sugar consumption were conducted with 162 children from Peru and 128 children from Spain. All other main analyses included 162 children from Peru and 129 children from Spain. For between-group comparisons of continuous variables, effect sizes were estimated as Cohen’s d (with the corresponding 95% confidence interval) for parametric comparisons and as the rank-biserial correlation for the Mann–Whitney U test; for categorical comparisons, prevalence differences were accompanied by 95% confidence intervals.

Distributional assumptions were assessed descriptively by inspecting histograms, Q–Q plots, and summary statistics. Because the caries score and behavioural variables were ordinal, bounded, or non-normally distributed, non-parametric tests were preferred for the main between-group comparisons of these variables. Welch’s *t*-test was used only as a descriptive comparison of mean OHI-S and mean sugar-consumption scores because equal variances could not be assumed. Toothbrushing frequency and sugar consumption were treated as ordinal variables in bivariate analyses. In the exploratory regression models, they were entered as ordered scores to describe linear trends, but this modelling choice is acknowledged as a limitation because the distances between categories cannot be assumed to be strictly equal. No adjustment for multiple comparisons was applied; all secondary comparisons and correlations should therefore be interpreted as exploratory.

Descriptive statistics were used to summarize the study variables: continuous variables were expressed as means with standard deviations (SDs) and as medians with interquartile ranges (IQRs), whereas categorical variables were expressed as frequencies and percentages. For the comparative analysis, continuous non-normally distributed variables were compared between the two groups using the Mann–Whitney U test, while the independent-samples *t*-test with Welch’s correction was applied where appropriate. Categorical variables were compared using the chi-square test or Fisher’s exact test, as appropriate.

Bivariate associations between OHI-S, toothbrushing frequency, sugar consumption, and the global caries score were examined within each group by calculating Spearman’s rank correlation coefficients. Finally, exploratory multiple linear regression was performed within each population to describe the joint associations of OHI-S, toothbrushing frequency, and sugar consumption with the global caries score. Because the caries score is a count-based outcome that may exhibit overdispersion, linear regression is suboptimal and these models are reported as exploratory descriptive analyses rather than as definitive inferential models; in particular, the coefficients should be understood as descriptive joint associations and not as fully adjusted causal estimates, because the models do not adjust for age, sex, dentition type, or school. Effect sizes for the regression coefficients are reported as unstandardised B coefficients with 95% confidence intervals. We did not fit a combined cross-country model with formal country-by-behaviour interaction terms because country, school, and rural/urban setting were structurally confounded in the present design; we acknowledge that, in a sufficiently powered and structurally comparable sample, Poisson or negative binomial regression with adjustment for age, sex, dentition type, and school—and with interaction terms—would provide a more appropriate analytical framework, and we recommend this strategy for future studies.

### 2.7. Ethical Considerations

The study protocol was approved by the Research Ethics Committee of the Universidad Católica de Valencia (Project Code: UCV/2024–2025/101). The Spanish Research Ethics Committee approval explicitly covered the entire multicentre protocol, including the Peruvian Amazon study site, after review of the local conditions of fieldwork. Because the study was conducted in a rural school setting in the Loreto region, where no accredited Peruvian research ethics committee was available within reasonable geographic reach, additional local authorisation was obtained from the regional educational authorities, the school principal, and the recognised community leaders of the participating Amazonian community, following standard practice for non-interventional school-based oral health studies in this setting. We acknowledge as a limitation that an independent review by an accredited Peruvian research ethics committee was not obtained, and we recommend that future studies in the region include such a review when feasible. Participation was voluntary, and written informed consent was obtained from parents or legal guardians prior to enrollment. Children were adequately informed about the study procedures and participated under assent conditions. Consent and assent information was provided in Spanish, which was the language used for school communication in the participating centres. No directly identifying information was transferred outside the local field records. The anonymised analytic dataset was transferred to and stored at the Universidad Católica de Valencia using password-protected files accessible only to the research team. All data were anonymized prior to analysis. Personal data were stored separately and processed in compliance with European data protection regulations (GDPR) and applicable Peruvian data protection laws.

## 3. Results

### 3.1. Characteristics of the Study Population

No statistically significant between-group differences were detected in age or sex distribution (Table 1).

The single-year age distribution was as follows for Peru and Spain, respectively: age 5 years, 1 vs. 0 children; 6 years, 30 vs. 22; 7 years, 22 vs. 20; 8 years, 20 vs. 23; 9 years, 33 vs. 16; 10 years, 12 vs. 21; 11 years, 35 vs. 25; and 12 years, 9 vs. 2. These differences should be considered when interpreting the unadjusted caries comparisons.

### 3.2. Dental Caries Experience

No statistically significant difference in mean global caries score or in caries prevalence was detected between the two populations (Table 2). This finding is interpreted as the absence of a detectable between-group difference under the available sample size, and not as evidence of equivalent caries burden. The mean difference in global caries score (Spain − Peru) was 0.36 points (95% CI: −0.38 to 1.10), which does not exclude clinically meaningful differences in either direction (Cohen’s d = 0.12, 95% CI: −0.12 to 0.35). Caries prevalence was 84.0% in Peru and 76.0% in Spain; the prevalence difference (Peru − Spain) was 8.0 percentage points (95% CI: −1.3 to 17.3), indicating that the observed difference was imprecise and should not be interpreted as evidence of equivalence.

Beyond the comparable mean scores, the distribution of caries experience differed between groups. For descriptive purposes only, and without treating these thresholds as validated diagnostic cut-offs for mixed dentition, a global caries score of 5–7 was labelled as moderate caries burden and a score ≥ 8 as severe caries experience. Under this descriptive classification, a moderate caries burden (score 5–7) was observed in 25.9% of the Peruvian children and 20.2% of the Valencian children, whereas severe caries experience (score ≥ 8) was more frequent in the Valencian group than in the Peruvian group (21.7% vs. 10.5%; *p* = 0.014). It is also worth clarifying the apparent inconsistency observed between the two indicators in Table 2: caries prevalence (any score > 0) was numerically higher in the Peruvian group (84.0% vs. 76.0%), whereas the mean global caries score was numerically lower (3.71 vs. 4.07). This pattern is internally coherent and is explained by the right-tail distribution of the caries score, which carried more weight in the Valencian group. In other words, caries affected a slightly larger proportion of Peruvian children, but severe caries experience was more common among Valencian children. The difference in mean global caries score did not reach statistical significance.

### 3.3. Oral Hygiene Status

Peruvian children showed significantly lower (better) mean OHI-S scores than Valencian children, and the distribution across hygiene categories also differed between groups (Table 3).

The difference in the distribution of hygiene categories was also significant, driven by fewer Peruvian children in the “poor” category and more in the “fair” category relative to the Valencian group.

### 3.4. Toothbrushing Frequency

Toothbrushing frequency was higher in the Peruvian group, both in the ordinal distribution and in the proportion of children brushing at least twice daily (Table 4). Because the upper category was open-ended (“three or more times daily”), the median, interquartile range, and category distribution should be prioritised over the arithmetic mean.

**Figure 1:**
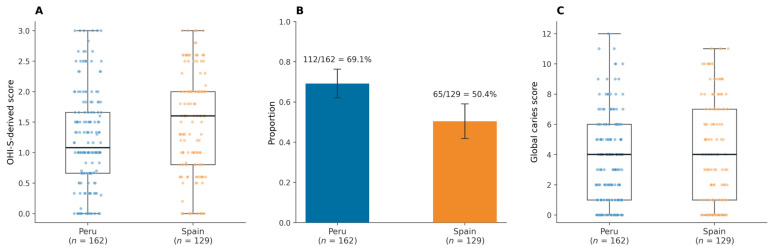
Distribution of oral hygiene, toothbrushing frequency, and global caries score by study group. (**A**) OHI-S-derived oral hygiene score by study group, displayed as boxplots with individual observations; lower values indicate better oral hygiene. (**B**) Proportion of children brushing at least twice daily, with 95% confidence intervals. (**C**) Global caries score by study group, displayed as boxplots with individual observations. The figure is intended as a descriptive visual summary of the participating schools and should not be interpreted as evidence of equivalent caries burden or causal effects of hygiene behaviours.

The gap was most evident at the upper end of the scale: 69.1% of the Peruvian children brushed at least twice daily, compared with 50.4% of the Valencian children. The contrast between the better oral hygiene indicators and the more frequent toothbrushing observed in the Peruvian group and the absence of a statistically significant difference in caries experience between groups is summarised descriptively in Figure 1; this observation is presented as a pattern requiring further investigation and not as evidence of equivalence of caries burden between the two populations.

### 3.5. Sugar Consumption

Sugar consumption was high in both populations and did not differ significantly, either in the mean consumption score or in the proportion of children reporting frequent (>1/day) intake (Table 5).

### 3.6. Correlation Analyses

Bivariate analyses showed statistically significant but weak associations between the clinical and behavioral variables within each group (Table 6). In both populations, poorer oral hygiene was weakly associated with a higher global caries score, and more frequent toothbrushing was weakly associated with a lower score. Sugar consumption was not directly correlated with the global caries score in either population. In the Peruvian group, sugar consumption was weakly correlated with poorer oral hygiene, whereas this association was not observed in the Valencian group. Given the small magnitude of the coefficients and the exploratory nature of the analyses, these correlations should be interpreted cautiously.

### 3.7. Multivariate Analysis

In the exploratory multivariable linear regression models, the predictors accounted for only a modest proportion of the variance in the global caries score in both populations (Table 7). In the Peruvian group, poorer oral hygiene status and lower toothbrushing frequency were significantly associated with higher global caries scores, whereas sugar consumption was not significant. In the Valencian group, toothbrushing frequency remained significantly associated with the global caries score, while OHI-S and sugar consumption did not reach statistical significance. Given the count-based nature of the outcome, the absence of adjustment for age, sex, dentition type, and school, and the modest explanatory capacity of the models, these findings should be interpreted as exploratory joint associations rather than as independent causal predictors.

## 4. Discussion

### 4.1. Key Findings: Better Oral Hygiene Without a Corresponding Reduction in Caries Experience

The principal observation from the present study is that, among the participating schools, children from the Peruvian Amazon showed significantly better measured oral hygiene—including lower OHI-S-derived scores and more frequent toothbrushing—than Spanish children from disadvantaged urban schools, yet no statistically significant difference in mean global caries score was detected between the two groups. However, the distribution of caries was not identical: severe caries experience was more frequent among Valencian children. This descriptive pattern is consistent with the hypothesis that structural and contextual factors may condition the effect of individual hygiene behaviours in disadvantaged settings, but it should not be interpreted as a confirmed mechanism. The absence of a between-group difference in mean caries scores may reflect a genuinely similar average burden, limited statistical power, residual confounding, differences in the severity distribution, or unmeasured contextual exposures, and the present study cannot adjudicate between these explanations.

These findings align with the broader conceptual framework proposed by Peres et al. in the Lancet Series on Oral Health, which positions dental caries as a disease shaped by social, commercial, and structural determinants rather than individual behaviors alone [2]. Our results are consistent with this perspective by showing that current measured hygiene indicators were not accompanied by a lower cumulative caries score at the population-comparison level, although the temporal mismatch between current behaviours and lifetime caries experience precludes causal interpretation.

### 4.2. Absence of Expected Caries Differentials

The absence of a statistically significant difference in mean global caries score between the two study groups (3.71 vs. 4.07; *p* = 0.596) should be interpreted cautiously, given the exploratory design, limited statistical power, and the composite nature of the outcome. The previous literature has generally reported substantially higher caries prevalence in LMICs compared with high-income countries [6,12]. However, several factors may explain this finding.

First, both study groups were deliberately selected from schools officially classified as serving socioeconomically disadvantaged communities, although individual-level socioeconomic comparability could not be verified. The “singular schools” in Valencia serve populations at high risk of social exclusion, including immigrant communities and families experiencing multidimensional poverty [16]. Previous research conducted in similar Valencian schools reported a caries prevalence of 81.87% with a mean DMFT of 4.48 [16], figures remarkably consistent with our findings. This suggests that within-country socioeconomic gradients may be as pronounced as between-country differences.

Second, the absence of a detectable difference in mean global caries score may partly reflect the particular vulnerability of the Spanish population selected. While Spain maintains a universal public healthcare system, dental care for children is not fully integrated into primary care, and significant barriers to access persist among marginalized populations [24]. Studies among Roma children in Spain have documented caries levels comparable to those in developing countries, with DMF indices exceeding 4.5 and substantial unmet treatment needs [24].

### 4.3. Superior Hygiene Practices in the Peruvian Context

The finding that Peruvian children demonstrated better oral hygiene indicators than their Spanish counterparts is noteworthy but should be interpreted cautiously. Because school-level oral-health programmes, parental oral-health literacy, fluoride exposure, availability of hygiene materials, and supervised brushing activities were not systematically measured, the present study cannot determine why the Peruvian group showed lower OHI-S-derived scores and more frequent toothbrushing. Possible explanations include differences in school context, community practices, prior exposure to health-promotion activities [25], measurement variability, or unmeasured family-level factors, but these remain hypotheses. Future studies should collect direct information on school-based oral-health programmes, parental supervision, fluoride toothpaste use, and access to preventive dental services to test these mechanisms.

### 4.4. The Role of Structural Healthcare Factors

Within each population, individual hygiene behaviours were associated with caries variation in the expected direction; yet, between the two populations, the more favourable hygiene profile observed in the Peruvian group was not accompanied by a correspondingly lower caries score. One candidate explanation for this discordance—to be considered as a hypothesis rather than as a demonstrated mechanism—is that contextual and structural factors not measured in the present study may modulate the population-level relationship between hygiene practices and caries. None of the variables that would be required to test this hypothesis (community fluoride exposure, professional preventive services, dental attendance, availability of restorative care, parental dental health literacy, or supervised brushing programmes) was directly assessed. The following paragraphs therefore outline mechanisms that could plausibly contribute to the observed pattern, with the explicit caveat that the present design does not allow for these mechanisms to be confirmed or quantified.

Access to preventive and restorative dental services represents a key structural determinant that may help explain the comparable caries levels despite divergent hygiene practices. In Valencia, children have theoretical access to the Spanish public health system and professional dental care, although utilization barriers persist among marginalized populations [24]. In the Peruvian Amazon, geographic isolation and limited dental workforce may reduce access to professional services, meaning that even children with good hygiene who develop early carious lesions may have limited access to preventive treatments such as professional fluoride application, dental sealants, or prompt restorative interventions [15].

Fluoride exposure provides a useful example of how population-level prevention can modify caries risk beyond individual behaviors. The updated Cochrane review by Iheozor-Ejiofor et al. concluded that contemporary evidence suggests community water fluoridation may lead to only a small reduction in caries in children, with lower estimated effects than those reported in older studies conducted before widespread use of fluoride toothpaste [26]. Contemporary Australian data nevertheless support an association between lifetime exposure to fluoridated water and lower childhood caries experience after socioeconomic adjustment [27]. In the present study, fluoride exposure was not directly measured; therefore, its potential role should be considered as a contextual hypothesis rather than a demonstrated explanatory mechanism.

Israel’s experience provides an informative natural experiment: following the discontinuation of water fluoridation in 2014, Levy et al. reported that children exposed to fluoridated water during early childhood had lower caries-related treatment needs than those born after discontinuation, even after the introduction of free dental care legislation [28]. This finding supports the relevance of population-level prevention, although it should not be interpreted as direct evidence that structural factors always outweigh individual behaviors in all settings.

### 4.5. Behavioral Determinants Within Populations

The within-group analyses confirmed that oral hygiene and toothbrushing were related to caries experience, although the strength of these associations was modest. In the bivariate analyses, poorer oral hygiene was associated with higher caries scores in both populations, and more frequent toothbrushing was associated with lower caries scores. In the exploratory multivariable models, poorer oral hygiene and lower toothbrushing frequency remained associated with higher caries scores in the Peruvian group, whereas in the Valencian group only toothbrushing frequency remained statistically significant. These findings suggest that oral hygiene behaviours are relevant within each context, but they should not be interpreted as independent causal predictors because of the cross-sectional design, the count-based outcome, and the absence of adjustment for key covariates.

The observation that individual behaviors were associated with caries variation within populations, yet did not explain between-group patterns, may reflect unmeasured contextual, socioeconomic, service-related, or measurement factors. However, because these factors were not directly assessed, this interpretation remains hypothetical. Structural equation modelling studies have shown that socioeconomic status may influence caries through multiple pathways, including behaviours, access to care, environmental exposures, and psychosocial mechanisms [21,22], but the present study cannot determine the relative contribution of these pathways.

An important alternative interpretation that warrants explicit acknowledgement is reverse causation. Because the design is cross-sectional, the observed within-group associations between higher toothbrushing frequency or better OHI-S and lower caries scores cannot be assumed to reflect a protective effect of hygiene on caries. Children who have already experienced caries, dental pain, or restorative treatment may subsequently adopt more frequent and more effective toothbrushing in response to dental advice, parental concern, or the visible consequences of disease. Under this hypothesis, the cross-sectional behaviour–caries association would be partly driven by caries history influencing current hygiene practices, rather than by hygiene practices reducing current caries. Longitudinal studies that capture the temporal sequence of hygiene behaviours and caries onset are required to disentangle these directions of effect.

### 4.6. Dietary Factors

Sugar consumption was similarly high in both study populations and did not differ significantly between groups. This finding is consistent with the global nutrition transition, which has resulted in increased sugar consumption across LMICs [29,30]. The high prevalence of frequent sugar intake (~61–64%) in both groups likely represents a major shared risk factor that may have contributed to the comparable caries levels.

The lack of a significant correlation between sugar consumption and caries in either group may reflect the limitations of self-reported dietary data, which are subject to recall bias and social desirability effects. Alternatively, the nearly universal high sugar consumption in both populations may have resulted in insufficient variability to detect associations.

### 4.7. Implications for Public Health Policy

The findings of this study should be interpreted as hypothesis-generating rather than as evidence of intervention effectiveness. Oral hygiene promotion, toothbrushing with fluoride toothpaste, and reduction in frequent sugar intake remain core components of caries prevention. However, the observed pattern also highlights the need for future studies to measure broader contextual factors, including fluoride exposure, school-based preventive activities, dental attendance, availability of restorative care, and family-level socioeconomic conditions. In disadvantaged school settings, oral-health promotion is likely to require multilevel approaches, but the present cross-sectional study cannot determine whether structural interventions would be more effective than behavioural interventions. Therefore, specific policy options such as school dental programmes, fluoride-based prevention, outreach services, or integration of oral health into primary care should be considered as plausible areas for future evaluation rather than as interventions directly supported by the present data.

### 4.8. Limitations

This study has several limitations that should be acknowledged. First, the cross-sectional design precludes causal inference. A central limitation is the temporal mismatch between the exposures and the outcome: the global caries score reflects cumulative lifetime disease and treatment history, whereas OHI-S, toothbrushing frequency, and sugar consumption were measured at the time of examination. Current behaviours may therefore not represent past exposures during the period in which caries developed, and reverse causation is possible. Children who have already experienced caries, dental pain, or restorative treatment may have subsequently improved their brushing habits following dental advice or parental concern.

Second, no formal a priori sample-size calculation was performed. The post hoc power to detect the observed effect size for the between-group difference in global caries score (Cohen’s d = 0.12) was approximately 16%, and the minimum effect size detectable with 80% power for the available sample (*n* = 162 vs. *n* = 129; α = 0.05, two-sided) was approximately d = 0.33. The absence of a statistically significant between-group difference in caries score is therefore compatible with both genuinely similar burdens and meaningful undetected differences, and should not be interpreted as evidence of equivalence. Moreover, although the mean global caries score did not differ significantly between groups, severe caries experience was more frequent in the Valencian group; therefore, interpretation should consider both average caries burden and severity distribution.

Third, because only one rural Peruvian school and two urban Valencian schools were included, country, school, urban/rural setting, and local healthcare context are structurally confounded. The participating schools were purposively selected, no random sampling frame was constructed, and school-level contextual variables such as water source, fluoridation status, existing oral-health programmes, recent preventive campaigns, and routine access to dental services were not systematically recorded. In addition, observations were clustered within only three schools. Because the number of clusters was too small to support a reliable multilevel model, clustering by school could not be formally modelled; consequently, standard errors may be underestimated and school-level contextual effects cannot be separated from individual-level associations. Findings should therefore not be generalised beyond the participating schools.

Fourth, individual-level socioeconomic indicators, including household income, parental education, housing conditions, migration status, dental insurance, previous dental attendance, and dental service utilisation, were not collected. Therefore, individual-level socioeconomic comparability between the two populations could not be empirically verified, even though both groups were classified as disadvantaged by their respective national education authorities.

Fifth, the global caries score combines dmft and DMFT across primary, mixed, and permanent dentitions, which may obscure age- and dentition-specific patterns and conflate untreated and treated disease. Reporting separate dmft and DMFT estimates stratified by age was not possible because dentition-type data were available only for the Peruvian sample. The decomposed dmft and DMFT components (decayed, missing, and filled teeth) were also not consistently available for both populations, although in disadvantaged settings these components may carry different meanings regarding untreated disease and access to care. Better access to restorative care could shift the D/M/F composition without necessarily reducing the total caries score; therefore, the present outcome cannot directly test service-access mechanisms.

Sixth, behavioural exposure was operationalised by toothbrushing frequency only, without information on fluoride toothpaste use, fluoride concentration, parental supervision, brushing duration, brushing technique, timing of brushing, or interdental cleaning. Sugar exposure relied on a brief ordinal questionnaire and a single 24 h recall, which may not adequately reflect habitual intake or cumulative free-sugar exposure. The source of behavioural information (child-only versus parent-assisted response) was not systematically recorded, which may have introduced differential information bias between sites. In addition, one Valencian participant had missing sugar-consumption data, so analyses involving sugar consumption were conducted with a slightly smaller Spanish sample.

Seventh, dental examinations were conducted without radiographs, potentially underestimating proximal caries, and teeth were not professionally cleaned or air-dried before examination. Examiner calibration was performed before fieldwork, but formal blinding to behavioural questionnaire responses and formal longitudinal drift assessment during the full fieldwork period were not documented.

Eighth, the analytical strategy did not adjust between-group comparisons for age, sex, dentition type, or school, did not test interaction terms between country and behavioural variables in a combined model, and did not employ Poisson or negative-binomial regression with full covariate adjustment, which would have been more appropriate for a count-based outcome. The exploratory linear models explained only a modest proportion of the variance and should be interpreted as descriptive joint associations rather than as independent causal predictors.

Finally, although the Spanish Research Ethics Committee approval covered the multicentre protocol including the Peruvian Amazon site, independent review by an accredited Peruvian research ethics committee was not obtained for this study and is recommended for future fieldwork in the region.

## 5. Conclusions

Among disadvantaged children attending the three participating schools, those from the rural Peruvian Amazon school showed significantly better oral hygiene and more frequent toothbrushing than those from two urban schools in Valencia, Spain, while no statistically significant difference in mean global caries score was detected between the two groups. However, severe caries experience was more frequent among Valencian children, indicating that average caries scores and severity distribution should be interpreted together. Within each population, poorer oral hygiene and lower toothbrushing frequency were associated with higher caries scores in bivariate analyses, although the exploratory multivariable models showed a more limited pattern of association. Because structural variables such as fluoride exposure, dental attendance, and access to preventive and restorative care were not measured, and because country, school, and rural/urban setting were structurally confounded in the present design, these findings should be regarded as descriptive observations within the participating schools rather than as evidence that structural factors outweigh behavioural interventions. Adequately powered longitudinal and multicentre studies, with individual-level socioeconomic data, dentition-stratified caries indices, direct measurement of structural exposures, and appropriate count-data models, are needed to test the contextual hypotheses suggested by the present comparison.

## Figures and Tables

**Table 1:** Sociodemographic characteristics of the study population.

Characteristic	Peru (*n* = 162)	Spain (*n* = 129)	*p*-Value
Age, years			
Mean ± SD	8.7 ± 1.9	8.6 ± 1.8	0.648 ^a^
Range	5–12	6–12	—
Sex, *n* (%)			
Male	77 (47.5%)	62 (48.1%)	0.928 ^b^
Female	85 (52.5%)	67 (51.9%)	

SD, standard deviation. ^a^ Mann–Whitney U test; ^b^ chi-square (χ^2^) test.

**Table 2:** Dental caries experience by study group.

Variable	Peru (*n* = 162)	Spain (*n* = 129)	*p*-Value
Global caries score			
Mean ± SD	3.71 ± 2.86	4.07 ± 3.44	0.596 ^a^
Median (IQR)	4.00 (1.00–6.00)	4.00 (1.00–7.00)	
Range	0–12	0–11	
Caries prevalence (global caries score > 0), %	84.0%	76.0%	0.120 ^b^

SD, standard deviation; IQR, interquartile range. Median (IQR) and range are descriptive. ^a^ Mann–Whitney U test; ^b^ chi-square (χ^2^) test.

**Table 3:** Oral hygiene status by study group.

Variable	Peru (*n* = 162)	Spain (*n* = 129)	*p*-Value
OHI-S-derived score (mean ± SD)	1.25 ± 0.79	1.49 ± 0.84	0.014 ^a^
Median (IQR)	1.08 (0.66–1.66)	1.60 (0.80–2.00)	
OHI-S-derived category, *n* (%)			
Good (0.0–0.6)	32 (19.8%)	28 (21.7%)	0.032 ^b^
Fair (0.7–1.8)	90 (55.6%)	53 (41.1%)	
Poor (1.9–3.0)	40 (24.7%)	48 (37.2%)	

SD, standard deviation; IQR, interquartile range. ^a^ Welch’s *t*-test (mean OHI-S); ^b^ chi-square (χ^2^) test (OHI-S category distribution).

**Table 4:** Toothbrushing frequency by study group.

Brushing Frequency	Peru (*n* = 162)	Spain (*n* = 129)	*p*-Value
Mean ± SD	1.90 ± 0.74	1.57 ± 0.86	<0.001 ^a^
Median (IQR)	2.0 (1.0–2.0)	1.0 (1.0–2.0)	
Distribution, *n* (%)			
Never (0)	2 (1.2%)	12 (9.3%)	<0.001 ^b^
Once daily (1)	48 (29.6%)	52 (40.3%)	
Twice daily (2)	77 (47.5%)	45 (34.9%)	
Three+ times daily (3)	35 (21.6%)	20 (15.5%)	

SD, standard deviation. ^a^ Mann–Whitney U test (mean brushing frequency); ^b^ chi-square (χ^2^) test (frequency distribution).

**Table 5:** Sugar consumption by study group.

Variable	Peru (*n* = 162)	Spain (*n* = 128)	*p*-Value
Mean ± SD	1.61 ± 0.49	1.64 ± 0.48	0.607 ^a^
Median (IQR)	2.0 (1.0–2.0)	2.0 (1.0–2.0)	
High consumption (>1/day), %	61.1%	64.1%	0.606 ^b^

SD, standard deviation; IQR, interquartile range. Sugar consumption was coded as 1 = up to one sugar-containing food or beverage per usual day (≤1/day) and 2 = more than one sugar-containing food or beverage per usual day (>1/day). ^a^ Welch’s *t*-test (mean consumption score); ^b^ chi-square (χ^2^) test (proportion with high consumption).

**Table 6:** Spearman correlation coefficients between study variables.

Variable Pair	Peru (ρ, *p*-Value)	Spain (ρ, *p*-Value)
OHI-S vs. global caries score	0.246, 0.002	0.208, 0.018
Brushing vs. global caries score	−0.169, 0.032	−0.214, 0.015
OHI-S vs. sugar consumption	0.253, 0.001	0.117, 0.194
Brushing vs. OHI-S	−0.024, 0.771	−0.196, 0.027
Brushing vs. sugar consumption	0.013, 0.879	−0.100, 0.267
Sugar consumption vs. global caries score	0.110, 0.169	−0.095, 0.283

ρ, Spearman’s rank correlation coefficient; *p*-values from the two-tailed Spearman correlation test. Peru, *n* = 162; Spain, *n* = 129, except for correlations involving sugar consumption in the Spanish group, which were calculated with *n* = 128 because one participant had missing sugar-consumption data. Statistical significance set at *p* < 0.05.

**Table 7:** Exploratory multiple linear regression models predicting the global caries score.

Variable	Peru (B, *p*-Value)	Spain (B, *p*-Value)
OHI-S	0.773, 0.008	0.632, 0.077
Toothbrushing frequency	−0.669, 0.023	−0.755, 0.031
Sugar consumption	0.320, 0.487	−1.034, 0.094
Model R^2^	0.087	0.084

B, unstandardized regression coefficient; R^2^, coefficient of determination. Multiple linear regression with the global caries score as the dependent variable; OHI-S, toothbrushing frequency, and sugar consumption entered simultaneously. Peru, *n* = 162; Spain, *n* = 128 for the model including sugar consumption. Standard errors (SEs) and 95% confidence intervals (95% CIs) for each unstandardised coefficient were as follows. Peru—OHI-S: SE = 0.286, 95% CI 0.209 to 1.338; toothbrushing frequency: SE = 0.292, 95% CI −1.246 to −0.093; sugar consumption: SE = 0.460, 95% CI −0.588 to 1.228. Spain—OHI-S: SE = 0.354, 95% CI −0.068 to 1.332; toothbrushing frequency: SE = 0.346, 95% CI −1.439 to −0.070; sugar consumption: SE = 0.613, 95% CI −2.248 to 0.179. Models did not adjust for age, sex, dentition type, or school. Diagnostic checks of the count-based outcome indicated overdispersion, supporting the recommendation that Poisson or negative-binomial regression with full covariate adjustment be used in confirmatory analyses. The models presented here should therefore be regarded as exploratory descriptive analyses.

## Data Availability

The anonymised analytic dataset, variable dictionary, and analysis syntax are available from the corresponding author upon reasonable request, subject to compliance with applicable data protection regulations. The data are not publicly deposited because the dataset derives from school-based research involving minors in socioeconomically vulnerable communities.

## Supplementary Materials

The following supporting information can be downloaded at https://www.mdpi.com/article/10.3390/dj14070459/s1, Supplementary File S1: Data Collection Instruments, Operational Definitions, and Examiner Calibration Protocol [31].

## Abbreviations

The following abbreviations are used in this manuscript:

CAOD	Cariados, ausentes, obturados (caries index for permanent dentition; Spanish equivalent of the DMFT index)
cod	Lower-case caries index applied to the primary (deciduous) dentition
DMF	Decayed, missing, and filled (teeth) index
DMFT	Decayed, missing, and filled teeth
GDPR	General Data Protection Regulation
IQR	Interquartile range
LED	Light-emitting diode
LMICs	Low- and middle-income countries
OHI-S	Simplified Oral Hygiene Index
SD	Standard deviation
SPSS	Statistical Package for the Social Sciences
STROBE	Strengthening the Reporting of Observational Studies in Epidemiology
WHO	World Health Organization
