# Supplementary material for: Oral Health Status, Oral Hygiene, and Behavioural Factors Among Disadvantaged Children: A Cross-Sectional Comparison Between the Peruvian Amazon and Valencia, Spain
Source: Dent J (Basel). 2026 Jul 21;14(7):459. doi: 10.3390/dj14070459 (PMC13407464; doi:10.3390/dj14070459)
Supplement: Supplementary file 1 [file dentistry-14-00459-s001.zip › dentistry-4413464-supplementary.pdf]

Supplementary File S1

Data Collection Instruments, Operational Definitions, and Examiner Calibration Protocol

"Oral Health Status, Oral Hygiene, and Behavioural Factors Among Disadvantaged Children: A Cross-Sectional Comparison Between the Peruvian Amazon and Valencia, Spain"

Miralles-Jordá L., Espinosa-Giménez J., Paradowska-Stolarz A., Fernández-Mafé M., Gómez-Adrián M.D., Murillo-Llorente M.T., Pérez-Bermejo M.*, Legidos-García M.E.

** Correspondence: marcelino.perez@ucv.es*

Contents

**S1.1**	Overview and study workflow	3
**S1.2**	Sociodemographic and clinical record sheet	4
**S1.3**	Dental caries assessment (dmft / DMFT) — criteria and recording sheet	5
**S1.4**	Oral hygiene assessment and OHI-S-derived score — criteria, calculation, and recording sheet	7
**S1.5**	Toothbrushing frequency questionnaire	10
**S1.6**	Sugar consumption frequency questionnaire and ordinal scoring system	11
**S1.7**	24-hour dietary recall (complementary instrument)	13
**S1.8**	Examiner training and calibration protocol	14
**S1.9**	Quality control and missing data handling	15
**S1.10**	STROBE checklist for cross-sectional studies	18

S1.1. Overview and study workflow

This file documents the instruments, operational definitions, and quality-control procedures used to collect the data analysed in the manuscript. The same set of instruments was used in both study sites (one rural school in the Peruvian Amazon and two urban "singular schools" in Valencia, Spain). All forms were administered in Spanish; the present file provides the working English translation used by the bilingual investigator team, with the original Spanish data collection sheet reproduced in Appendix A.

Timeline

- **Peru (Requena province, Loreto region):** clinical examinations from April 2025 to July 2025.
- **Spain (Valencia, Colegio Madre Petra and Colegio Santiago Apóstol):** clinical examinations from September 2025 to October 2025.
- **Behavioural and dietary data:** collected on the same day as the clinical examination.

Setting and equipment

Examinations were performed in school facilities (multipurpose rooms or empty classrooms) made available for the study. No portable dental unit was used. Each child was examined seated in an upright position. All clinical examinations were conducted under standardized conditions using:

- LED examination headlamp (portable, dental-grade, neutral white light).
- Sterile flat dental mirrors (No. 5), one per child.
- WHO Community Periodontal Index (CPI) periodontal probes, used for tactile confirmation of cavitated lesions only.
- Disposable gauze and cotton rolls.
- Personal protective equipment: gloves, masks, and protective eyewear for the examiner.
- Hand hygiene and instrument disinfection between children, in accordance with standard cross-infection control.

No radiographic examinations were performed; this limitation is acknowledged in the manuscript.

Examiner team

Clinical examinations were performed by two calibrated dental examiners (one operating in each site), trained together before fieldwork against a reference examiner (a senior researcher with previous epidemiological experience). Each examination day, one assistant recorded the findings dictated by the examiner on the data collection sheet.

Informed consent

Written informed consent was obtained from parents or legal guardians before fieldwork. Children gave verbal assent on the day of examination. A child could withdraw assent at any point; in such cases, the examination was discontinued and the partial record was discarded.

S1.2. Sociodemographic and clinical record sheet

The following identification block headed every record. School identifier and child code allowed the link between the clinical record, the behavioural questionnaire, and the dietary recall while preserving anonymity (no names were stored in the analytic dataset).

Field	Specification / response options
**School identifier**	R = Requena (Peru) / P = Colegio Madre Petra (Valencia) / S = Colegio Santiago Apóstol (Valencia)
**Child code**	Sequential numeric code within school (e.g., R1, R2 … ; P1, P2 …)
**Age**	Completed years at the date of examination (range 5–12).
**Sex**	1 = male / 2 = female (self-reported, confirmed by school records).
**Dentition type**	1 = primary / 2 = mixed / 3 = permanent. Assigned by the examiner from the clinical examination. Recorded for the Peruvian sample only; not available at this level for the Valencian sample (see manuscript).
**Date of examination**	DD/MM/YYYY.
**Examiner**	Examiner code (E1 or E2).

Note. *Individual-level socioeconomic data (household income, parental education, dental insurance, dental-care access) were not collected. School-level classification by the respective national education authorities was used as a proxy for socioeconomic disadvantage. This is acknowledged as a limitation in the main manuscript.*

S1.3. Dental caries assessment (dmft / DMFT)

S1.3.1. Diagnostic criteria

Caries detection followed the standardized WHO criteria for epidemiological oral health surveys (Oral Health Surveys – Basic Methods, 5th ed., World Health Organization, Geneva, 2013). A tooth surface was scored as decayed only if a frank cavitated lesion or a clearly softened floor / undermined enamel could be detected with the dental mirror, supported by tactile confirmation with the WHO/CPI probe when needed. Doubtful or non-cavitated white-spot lesions were not recorded as decayed.

Permanent	Primary	Operational definition
**D**	**d**	Decayed: cavitated carious lesion clinically detectable on any surface of the tooth.
**M**	**m**	Missing due to caries: permanent tooth absent because of caries (M) / primary tooth lost before the expected age of exfoliation due to caries (m). Teeth missing for other reasons (e.g., orthodontics, congenital absence, normal exfoliation in primary dentition) were not scored.
**F**	**f**	Filled: tooth with one or more restorations because of caries and no current cavitated lesion.
—	—	Sound: tooth present, no caries, no restorations. Not counted in the index.

S1.3.2. Indices

dmft (primary dentition): sum of decayed, missing (due to caries), and filled primary teeth for the child.

DMFT (permanent dentition): sum of decayed, missing (due to caries), and filled permanent teeth for the child. CAOD (“cariados, ausentes y obturados – dientes”) is the Spanish-language equivalent term used in the original data collection sheet.

Global caries score: for children in mixed dentition, the sum of dmft and DMFT was used as a single composite “global caries score”. The methodological caveats of this composite (different number of teeth at risk, different susceptibility, conflation of treated and untreated disease across age groups) are discussed in the main manuscript.

S1.3.3. Tooth-level recording grid (FDI two-digit notation)

Each tooth was scored on the grid below using S (sound), D/d, M/m, or F/f. The grid follows the FDI two-digit notation. For each tooth, only the worst status was recorded (D/d > F/f > sound).

Permanent dentition

18	17	16	15	14	13	12	11	21	22	23	24	25	26	27	28
48	47	46	45	44	43	42	41	31	32	33	34	35	36	37	38

Cells under each tooth number are filled with S, D, M, or F.

Primary dentition

55	54	53	52	51
85	84	83	82	81

61	62	63	64	65
71	72	73	74	75

Cells under each tooth number are filled with s (sound), d (decayed), m (missing due to caries), or f (filled).

S1.3.4. Per-child summary

Index	Decayed	Missing	Filled
dmft (primary)			
DMFT (permanent)			
Global caries score (dmft + DMFT)			

S1.4. Oral hygiene assessment and OHI-S-derived score

Oral hygiene was assessed using selected index tooth surfaces based on the Greene and Vermillion framework (J Am Dent Assoc 1964;68:7–13). For the analyses reported in the manuscript, an OHI-S-derived oral hygiene score ranging from 0 to 3 was used, with lower values indicating better oral hygiene. This score was obtained by averaging the debris/calculus scoring information recorded on the selected index surfaces and was then categorised as good, fair, or poor using the thresholds shown below.

S1.4.1. Index teeth and surfaces

Six tooth surfaces are scored per child: four buccal surfaces (upper first molars, upper right central incisor, lower left central incisor) and two lingual surfaces (lower first molars). When a permanent index tooth was not yet erupted (typical in primary or mixed dentition), the corresponding primary tooth was substituted (see substitution scheme).

Quadrant / position	Permanent (FDI)	Primary substitute (FDI)
Upper right — first molar (buccal)	**16**	55
Upper right — central incisor (labial)	**11**	51
Upper left — first molar (buccal)	**26**	65
Lower left — first molar (lingual)	**36**	75
Lower left — central incisor (labial)	**31**	71
Lower right — first molar (lingual)	**46**	85

If no permanent or primary substitute was available for an index position (e.g., extracted, severely destroyed), the position was not scored, and the OHI-S was calculated over the remaining valid surfaces (minimum 4 surfaces; otherwise the OHI-S was not computed for that child).

S1.4.2. Debris Index – Simplified (DI-S) criteria

Score	Criterion
**0**	No soft debris or stain present.
**1**	Soft debris covering not more than one third of the tooth surface, or presence of extrinsic stain without other debris, regardless of surface area.
**2**	Soft debris covering more than one third but not more than two thirds of the exposed tooth surface.
**3**	Soft debris covering more than two thirds of the exposed tooth surface.

S1.4.3. Calculus Index – Simplified (CI-S) criteria

Score	Criterion
**0**	No calculus present.
**1**	Supragingival calculus covering not more than one third of the exposed tooth surface.
**2**	Supragingival calculus covering more than one third but not more than two thirds of the exposed tooth surface, or presence of individual flecks of subgingival calculus around the cervical portion of the tooth.
**3**	Supragingival calculus covering more than two thirds of the exposed tooth surface, or a continuous heavy band of subgingival calculus around the cervical portion of the tooth.

S1.4.4. Calculation

DI-S = (sum of debris scores on the valid index surfaces) ÷ (number of surfaces scored).

CI-S = (sum of calculus scores on the valid index surfaces) ÷ (number of surfaces scored).

For the analyses reported in the manuscript, an OHI-S-derived oral hygiene score was calculated on a 0–3 scale as the mean of the debris/calculus information recorded for the valid index surfaces. Lower values indicate better oral hygiene.

This analytical score was used because the good/fair/poor categories applied in the field form and in the manuscript correspond to a 0–3 oral hygiene scale. The classical OHI-S total score, calculated as DI-S + CI-S and ranging from 0 to 6, was not used as the categorical analytical variable in the manuscript.

S1.4.5. Categorisation

OHI-S-derived score range	Category	Interpretation
0.0 – 0.6	**Good**	Adequate oral hygiene.
0.7 – 1.8	**Fair**	Intermediate level; preventive reinforcement recommended.
1.9 – 3.0	**Poor**	Inadequate hygiene; oral health education indicated.

S1.4.6. Recording grid

On the recording sheet, the examiner enters the DI-S and CI-S scores (0–3) on the corresponding surface of each index tooth. Substitute tooth numbers are pre-printed where applicable.

	**16**<br>/ 55<br>*buccal*	**11**<br>/ 51<br>*labial*	**26**<br>/ 65<br>*buccal*	**46**<br>/ 85<br>*lingual*	**31**<br>/ 71<br>*labial*	**36**<br>/ 75<br>*lingual*
**DI-S (0–3)**						
**CI-S (0–3)**						

	**DI-S**	**CI-S**	**OHI-S-derived score**
**Mean score / number of valid surfaces**			
**Category (good / fair / poor)**			

S1.5. Toothbrushing frequency questionnaire

Toothbrushing frequency was assessed by a brief structured interview administered to each child on the day of the clinical examination. When possible, responses were corroborated by the parent or guardian (notes section). The full questionnaire as administered is reproduced below.

Question administered (English working translation)

"How many times do you brush your teeth on a usual day?"

Original Spanish wording: *"¿Cuántas veces te cepillas los dientes en un día normal?"*

Response options and coding

Code	English	Spanish
**0**	Never / not on a usual day.	*Nunca / no en un día normal.*
**1**	Once a day.	*Una vez al día.*
**2**	Twice a day.	*Dos veces al día.*
**3**	Three or more times a day.	*Tres o más veces al día.*

Variables not captured by this instrument

The following dimensions of toothbrushing behaviour were NOT captured by the present questionnaire and are therefore not available in the analytic dataset:

- Use and concentration of fluoride toothpaste.
- Duration of each brushing episode.
- Brushing technique (e.g., Bass, Stillman, modified-roll, or unspecified).
- Parental supervision (presence, frequency, and quality of supervision).
- Use of dental floss, interdental brushes, or mouth rinses.
- Time of day of brushing (e.g., morning vs. bedtime).

This limitation is acknowledged in the main manuscript and is recommended for incorporation into future fieldwork in the same populations.

S1.6. Sugar consumption frequency questionnaire

Sugar consumption was assessed by a brief ordinal frequency questionnaire that focused on the habitual daily intake of clearly cariogenic sugar-containing foods and beverages, complemented by a 24-hour recall (Section S1.7) to support classification. The two instruments were always administered jointly.

S1.6.1. Foods and beverages considered

The following items were classified as “sugar-containing” for the purpose of this questionnaire:

Foods	Beverages
• Sweets, candies, lollipops, chewing gum with sugar<br>• Chocolate and chocolate spread<br>• Industrial bakery (croissants, doughnuts, pastries)<br>• Sweet biscuits and cookies<br>• Breakfast cereals with added sugar<br>• Sweetened yogurts and dairy desserts<br>• Ice cream and sorbets<br>• Jam, marmalade, honey, condensed milk<br>• Sweet local snacks (e.g., chancaca, dulces tradicionales)	• Carbonated soft drinks (cola, lemon, orange, etc.)<br>• Industrial fruit juices and nectars<br>• Powdered or syrup-based flavoured drinks (e.g., chicha morada with added sugar, sweetened lemonade)<br>• Sweetened milk drinks (chocolate milk, strawberry milk)<br>• Sweetened tea or coffee<br>• Energy drinks (rare in this age group)<br>• Sweetened yogurt drinks

Excluded items: fresh whole fruit, plain milk, plain yogurt, unsweetened water, and unsweetened infusions were *not* counted as sugar exposures in this questionnaire.

S1.6.2. Question administered

“On a usual day, how often do you eat or drink any of the foods or drinks shown in this list?”

Original Spanish wording: *“En un día normal, ¿con qué frecuencia tomas alguno de los alimentos o bebidas de esta lista?”*

The interviewer presented the list visually (printed sheet with simple pictograms for the youngest children) and recorded the most frequent pattern reported for a usual day, corroborated against the 24-hour recall.

S1.6.3. Ordinal scoring system

Code	Category	Operational definition (occasions per usual day)
**1**	**Low / moderate**	Up to one sugar-containing food or beverage per usual day (≤1/day).
**2**	**High**	More than one sugar-containing food or beverage per usual day (>1/day).

Cut-off note. *The "high consumption (>1/day)" threshold reported in the main manuscript (Table 5) corresponds to category 2 of this ordinal scale. The two-level operational scale used for the present analysis is a simplification of a richer FFQ that was not feasible to deploy in the field conditions of the rural Amazonian site, and it represents a conservative measure of cariogenic dietary exposure.*

S1.6.4. Limitations of this instrument

- The questionnaire records frequency, not portion size or absolute sugar load.
- It does not differentiate between intra-meal and between-meal sugar exposures (the latter being more cariogenic).
- It does not capture the time of day of sugar intake (e.g., bedtime exposure).
- It relies on self-report and is therefore subject to recall and social-desirability bias.
- With only two ordinal categories in the analytic dataset, variability is limited and small associations may not be detectable; this is acknowledged in the manuscript.

S1.7. 24-hour dietary recall (complementary instrument)

A child-adapted 24-hour recall was administered on the same day as the clinical examination, with the help of the parent or guardian whenever possible. Its purpose was to support the classification of the child in the ordinal sugar-consumption scale of Section S1.6, not to estimate absolute nutrient intake. Two registered dietitians supervised the procedure for cross-site consistency.

Form (as administered)

Eating occasion	Foods and drinks reported (free text)	Approximate portion	Sugar-containing? (yes / no)
**Breakfast**			
**Mid-morning snack**			
**Lunch**			
**Afternoon snack**			
**Dinner**			
**Before bedtime**			
**Other**			

Final summary: Total number of distinct sugar exposures during the preceding 24 hours: ______. This count is used jointly with the response to the structured frequency question (Section S1.6.2) to assign the child to category 1 (≤1/day) or category 2 (>1/day).

Discrepancies between the recall and the frequency answer were resolved through a brief follow-up probe with the child (and parent when available) to identify whether the recall day was atypical (e.g., birthday, school party, religious celebration). In such cases the structured frequency response was retained.

Methodological note

A single 24-hour recall, even when child-adapted and parent-supported, cannot fully capture habitual dietary intake. Its use here is restricted to supporting the ordinal classification of sugar exposure on a typical day; it was not used to estimate calorie or macronutrient intake. This limitation is acknowledged in the manuscript.

S1.8. Examiner training and calibration protocol

S1.8.1. Reference examiner and training

A senior dental researcher with previous experience in school-based oral health surveys acted as the gold-standard reference examiner. Before fieldwork, the two field examiners (E1 for Spain and E2 for Peru) underwent the following joint training:

- Two theoretical sessions on WHO caries criteria and Greene & Vermillion OHI-S criteria, with case-based discussion.
- One practical workshop using clinical photographs and study models to standardise threshold judgements (e.g., cavitated vs. non-cavitated lesions, debris coverage thresholds).
- One clinical pilot session in a paediatric dental clinic in Valencia (15 volunteer children outside the study), where examiners and reference examiner independently scored each child.

S1.8.2. Calibration design

Two types of agreement were quantified:

- **Intra-examiner reliability:** each field examiner re-examined approximately 10% of the children examined on the same day, blinded to the first record (re-examination conducted at the end of the same examination session).
- **Inter-examiner reliability:** during the pilot session, both field examiners and the reference examiner independently scored the same children; pairwise agreement was then computed.

Cohen's kappa coefficient was computed for each pair (examiner vs. self for intra-examiner; examiner vs. reference for inter-examiner) using cross-tabulations of the per-tooth (caries) or per-surface (OHI-S) classifications.

S1.8.3. Achieved agreement

Outcome	Intra-examiner kappa	Inter-examiner kappa
**Caries detection (per tooth)**	**0.91**	**0.86**
**OHI-S scoring (per surface)**	**0.88**	**0.82**

All values are above the conventional 0.80 threshold for "almost perfect" agreement (Landis & Koch, 1977). Inter-examiner kappa for OHI-S was the lowest value observed (0.82), driven by occasional borderline scores between debris categories 1 and 2; this was further mitigated during fieldwork by the use of pre-printed examples on the back of the data collection sheet.

S1.8.4. In-field quality control

- Each examination form was checked for completeness by the assistant before the child left the room.
- Forms with missing key variables (dmft/DMFT, OHI-S, brushing, sugar) were flagged for follow-up and, when correction was not possible, the affected child was excluded from the corresponding variable's analysis on a pairwise basis (see Section S1.9).
- Data entry was performed in duplicate by two researchers; discrepancies were resolved against the original paper form before lock.

S1.9. Quality control and missing data handling

S1.9.1. Eligibility flow

Step	Peru (rural school)	Valencia (two singular schools)
Children enrolled in the school during the data-collection period	**178**	**152**
Children whose parents/guardians did not return a signed consent form	12	18
Children excluded for incomplete clinical or behavioural data	4	5
**Children included in the analytic sample**	**162**	**129**
**Participation rate**	**91.0%**	**84.9%**

Note. The analytic sample comprised 162 children from Peru and 129 children from Valencia for the main clinical and behavioural variables. One additional missing value for sugar consumption was present in the Valencian group; therefore, analyses involving sugar consumption used n = 128 for Valencia.

S1.9.2. Missing data handling

Missing data were not imputed. After applying the exclusion criteria described above, one additional missing value remained for sugar consumption in the Valencian group. Therefore, analyses involving sugar consumption were conducted with 162 children from Peru and 128 children from Spain. All other main analyses included 162 children from Peru and 129 children from Spain. Complete-case analysis was used for each outcome or variable pair, and the per-variable sample size is reported in the relevant tables of the main manuscript. No child contributed data after withdrawal of assent.

S1.9.3. Data lock and access

The analytic dataset was locked once duplicate entry, range checks, and consistency checks were completed. The anonymised dataset and the original data collection forms are retained by the corresponding author (UCV – Universidad Católica de Valencia San Vicente Mártir) and are available on reasonable request, in compliance with European data protection regulations (GDPR) and applicable Peruvian data protection laws.

S1.10. STROBE checklist for cross-sectional studies

STROBE item	Recommendation	Location in manuscript / supplementary material
**1a**	Indicate the study design with a commonly used term in the title or abstract.	Title; Abstract, Methods
**1b**	Provide an informative and balanced summary of what was done and what was found.	Abstract
**2**	Explain the scientific background and rationale for the investigation.	Introduction, paragraphs 1–5
**3**	State specific objectives, including any prespecified hypotheses.	End of Introduction
**4**	Present key elements of study design early in the paper.	Section 2.1 Study Design
**5**	Describe the setting, locations, and relevant dates, including periods of recruitment, exposure, follow-up, and data collection.	Section 2.1 Study Design; Section 2.2 Study Population and Setting; Supplementary File S1, Section S1.1
**6a**	Give the eligibility criteria and the sources and methods of selection of participants.	Section 2.2 Study Population and Setting; Section 2.3 Inclusion and Exclusion Criteria; Supplementary File S1, Section S1.9.1
**6b**	For matched studies, give matching criteria and number of exposed and unexposed.	Not applicable; this was not a matched study.
**7**	Clearly define all outcomes, exposures, predictors, potential confounders, and effect modifiers. Give diagnostic criteria, if applicable.	Section 2.4 Variables and Measurement Instruments; Supplementary File S1, Sections S1.2–S1.7
**8**	For each variable of interest, give sources of data and details of methods of assessment. Describe comparability of assessment methods if more than one group exists.	Section 2.4 Variables and Measurement Instruments; Section 2.5 Data Collection Procedures; Supplementary File S1, Sections S1.1–S1.8
**9**	Describe any efforts to address potential sources of bias.	Section 2.5 Data Collection Procedures; Section 2.6 Statistical Analysis; Section 4.8 Limitations; Supplementary File S1, Sections S1.8–S1.9
**10**	Explain how the study size was arrived at.	Section 2.3 Inclusion and Exclusion Criteria; Section 4.8 Limitations
**11**	Explain how quantitative variables were handled in the analyses. If applicable, describe which groupings were chosen and why.	Section 2.4 Variables and Measurement Instruments; Section 2.6 Statistical Analysis; Section 3 Results; Supplementary File S1, Sections S1.3–S1.7
**12a**	Describe all statistical methods, including those used to control for confounding.	Section 2.6 Statistical Analysis
**12b**	Describe any methods used to examine subgroups and interactions.	Section 2.6 Statistical Analysis; Section 4.8 Limitations

**12c**	Explain how missing data were addressed.	Section 2.6 Statistical Analysis; Supplementary File S1, Section S1.9.2
**12d**	For cohort studies, describe how loss to follow-up was addressed.	Not applicable; cross-sectional study.
**12e**	Describe any sensitivity analyses.	Section 2.6 Statistical Analysis; Section 4.8 Limitations. No formal sensitivity analyses were performed.
**13a**	Report numbers of individuals at each stage of the study.	Section 2.3 Inclusion and Exclusion Criteria; Supplementary File S1, Section S1.9.1
**13b**	Give reasons for non-participation at each stage.	Section 2.3 Inclusion and Exclusion Criteria; Supplementary File S1, Section S1.9.1
**13c**	Consider use of a flow diagram.	Supplementary File S1, Section S1.9.1 provides the recruitment-to-analysis flow in tabular form.
**14a**	Give characteristics of study participants.	Section 3.1 Characteristics of the Study Population; Table 1
**14b**	Indicate the number of participants with missing data for each variable of interest.	Section 2.6 Statistical Analysis; Tables 5–7; Supplementary File S1, Section S1.9.2
**14c**	For cohort studies, summarise follow-up time.	Not applicable; cross-sectional study.
**15**	Report numbers of outcome events or summary measures.	Section 3.2 Dental Caries Experience; Tables 2–5
**16a**	Give unadjusted estimates and, if applicable, confounder-adjusted estimates and their precision.	Section 3 Results; Tables 1–7
**16b**	Report category boundaries when continuous variables were categorised.	Section 2.4 Variables and Measurement Instruments; Tables 3–5; Supplementary File S1, Sections S1.4–S1.6
**16c**	If relevant, consider translating estimates of relative risk into absolute risk.	Not applicable; no relative-risk model was fitted. Prevalence differences are reported descriptively where applicable.
**17**	Report other analyses done, such as subgroup analyses and interaction analyses.	Section 3.6 Correlation Analyses; Section 3.7 Multivariate Analysis; Section 4.8 Limitations
**18**	Summarise key results with reference to study objectives.	Section 4.1 Key Findings; Section 5 Conclusions
**19**	Discuss limitations, taking into account sources of potential bias or imprecision.	Section 4.8 Limitations
**20**	Give a cautious overall interpretation of results considering objectives, limitations, multiplicity, and evidence from similar studies.	Section 4 Discussion; Section 5 Conclusions
**21**	Discuss the generalisability of the study results.	Section 4.8 Limitations; Section 5 Conclusions
**22**	Give the source of funding and the role of funders.	Funding statement
